# The potential for carbon bio-sequestration in China’s paddy rice (*Oryza sativa* L.) as impacted by slag-based silicate fertilizer

**DOI:** 10.1038/srep17354

**Published:** 2015-12-01

**Authors:** Alin Song, Dongfeng Ning, Fenliang Fan, Zhaojun Li, Mary Provance-Bowley, Yongchao Liang

**Affiliations:** 1 Key Laboratory of Plant Nutrition and Fertilizer, Ministry of Agriculture, Institute of Agricultural Resources and Regional Planning, Chinese Academy of Agricultural Sciences, Beijing 100081, P.R. China; 2 Harsco Metals and Minerals, 359 North Pike Rd Sarver, PA 16055 USA; 3 Ministry of Education Key Laboratory of Environment Remediation and Ecological Health, College of Environmental & Resource Sciences, Zhejiang University, Hangzhou, 310058, China

## Abstract

Rice is a typical silicon-accumulating plant. Silicon (Si), deposited as phytoliths during plant growth, has been shown to occlude organic carbon, which may prove to have significant effects on the biogeochemical sequestration of atmospheric CO_2_. This study evaluated the effects of silicate fertilization on plant Si uptake and carbon bio-sequestration in field trials on China’s paddy soils. The results showed (1) Increased Si concentrations in rice straw with increasing application rates of silicate fertilizer; (2) Strong positive correlations between phytolith contents and straw SiO_2_ contents and between phytolith contents and phytolith-occluded carbon (PhytOC) contents in rice straw; (3) Positive correlations between the phytolith production flux and either the above-ground net primary productivity (ANPP) or the PhytOC production rates; (4) Increased plant PhytOC storage with increasing application rates of silicate fertilizer. The average above-ground PhytOC production rates during China’s rice production are estimated at 0.94 × 10^6^ tonnes CO_2_ yr^−1^ without silicate fertilizer additions. However, the potential exists to increase PhytOC levels to 1.16–2.17 × 10^6^ tonnes CO_2_ yr^−1^ with silicate fertilizer additions. Therefore, providing silicate fertilizer during rice production may serve as an effective tool in improving atmospheric CO_2_ sequestration in global rice production areas.

Human activities and industrialization have led to increasingly higher levels of carbon dioxide (CO_2_) in the atmosphere, with the resultant negative effects on global climate systems^1^. Current estimates of CO_2_ emissions amounted to 36 billion tonnes for the calendar year 2013^2^. These increasing concentrations of atmospheric CO_2_ have been implicated as major contributors to global climate change, hence, negatively impacting human and environmental health and safety^3^. Thus, sustainable methods for reducing and sequestering atmospheric CO_2_ are needed.

A promising biogeochemical means of reducing atmospheric CO_2_ is the occlusion of carbon within plant phytoliths, also known as silica phytoliths or plant opals. Silicon is stored in plants mainly in the form of phytoliths^4^. These silica phytoliths comprise noncrystalline silica minerals deposited within cells and cell walls of different plant organs when monosilicic acid [Si(OH)_4_], is taken up by plant roots and transported to the aboveground organs, where phytoliths form near evaporative surfaces by deposition and polymerization^5^,^6^. The silica phytoliths that are formed occlude some of the organic carbon that is extracted from atmospheric CO_2_ during photosynthesis, which is then deposited during plant growth^7^. When plant residues are returned to the soil, they gradually decompose releasing these phytoliths. However, Phytolith-occluded carbon (PhytOC) is relatively stable and can remain in the soil for long periods of time, being present in Tertiary and Late Cretaceous sediments^7^,^8^,^9^. This carbon fraction has been recognized as an important long-term terrestrial carbon (C) sink, sequestering about 1.5 billion tonnes of CO_2_ annually^10^.

Recently, phytolith-occluded carbon has been investigated in some plant species including grass^11^ and forest^12^ species, millet (*Setaria italica*)^13^, bamboo^10^, sugarcane (*Saccharum sinensis*)^14^ and rice (*Oryza sativa*)^8^. These previous trials have demonstrated the potential for long-term biogeochemical sequestration of atmospheric CO_2_ during plant phytolith production, with grass species’ phytoliths determined to be less degradable in soil, and over geological time, than phytoliths from leaves of forest species^6^,^15^.

Rice, a Si accumulating species, actively accumulates Si at tissue concentrations of 5% or more of net aboveground biomass^16^. In China, rice is a staple food crop, and China continues to lead the world in rice production, with outputs totaling 28% of the total global rice production in 2011^17^. It is estimated that the rice growing area in China is 18.5% of the world’s total tillable land area. Therefore, due to the large production area, high above-ground net primary productivity (ANPP), and high Si accumulation, paddy rice cropping systems may play an important role, not only in terrestrial production of phytoliths^8^, but also in the sequestration of atmospheric CO_2_.

Plant phytoliths, returned to the soil via crop residue at the end of each season, contribute to the pools of phytoliths in the soil’s upper layers, becoming an important component of soil structural systems^18^; Si depletion is known to occur on traditional paddy rice soils due to a continuous monoculture of high-yielding varieties under intensive cultivation practices^19^. Desplanques *et al.*^20^ estimate that 270 kg ha^−1^ of biogenic Si is removed each year from rice cultivation and that without additional Si fertilizer inputs, it would take only five years to exhaust the soils’ dissolved Si supplies from continued rice production in Camargue, France.

Destruction of global soils has become an important issue with global soil health issues gaining international attention, and 2015 being named the International Year of Soils by the 68^th^ United Nations General Assembly^21^. Agricultural soils are largely composed of silicate minerals, and many soils contain adequate amounts of total Si, but are naturally low in soluble (plant-available) Si. Crop removal of Si can exceed the slow weathering processes of silicate clay minerals resulting in reductions in the Si supplying capacity of soils. Additionally, crop management practices of applications of acidifying fertilizer materials may increase the levels of soluble silicon in soils, but the removal of silicon from soils without fertilizer replacements has been implicated in the destruction of our once productive global agricultural soils^22^,^23^,^24^. Therefore, soluble Si supplies may be a limiting factor for sustainable rice production in some regions^24^,^25^. Silicon amendments have been shown to decrease lodging, reduce stress, and increase yields during paddy rice production^26^,^27^. Therefore, application of Si fertilizers to degraded paddy soils is now recognized as a best management practice in increasing and sustaining rice yields, in some Southeast Asian areas^26^.

Although an increasing body of evidence has shown the long-term C sequestration potential of silica phytoliths within soils^1^,^8^,^10^,^11^,^12^,^13^,^14^,^28^, the majority of these trials have been conducted in native ecosystems, and not under high production agriculture. Huang *et al*. provided the first field evidence that storage of PhytOC, within intensively cultivated soils, could be increased by heavy mulching of bamboo and rice residues, post-phytolith production^29^. However, the aim of the current research was to determine if phytolith production *in-planta* could be increased from the additions of supplemental silicate fertilizers, thus affecting long-term storage of C during biomass accumulation in intensive rice crop production. Therefore, research is still lacking on the effects of silicate fertilization on phytolith formation and biogeochemical sequestration under China’s rice paddy production systems.

To evaluate the C occlusion within phytoliths during rice cropping as affected by silicate fertilizer additions, in this study, we took rice straw samples at five sites receiving silicate fertilizer additions in southern China at rice grain maturity in 2012. Using the phytolith content-biogenic silica content transfer function obtained from the samples, and by applying grassland^11^ formulas for phytolith production flux and above-ground net primary productivity (ANPP), we arrived at an estimated value for the production of phytoliths and PhytOC in China’s paddy field ecosystems.

## Results

### Soil available silicon contents

**Figure 1:**
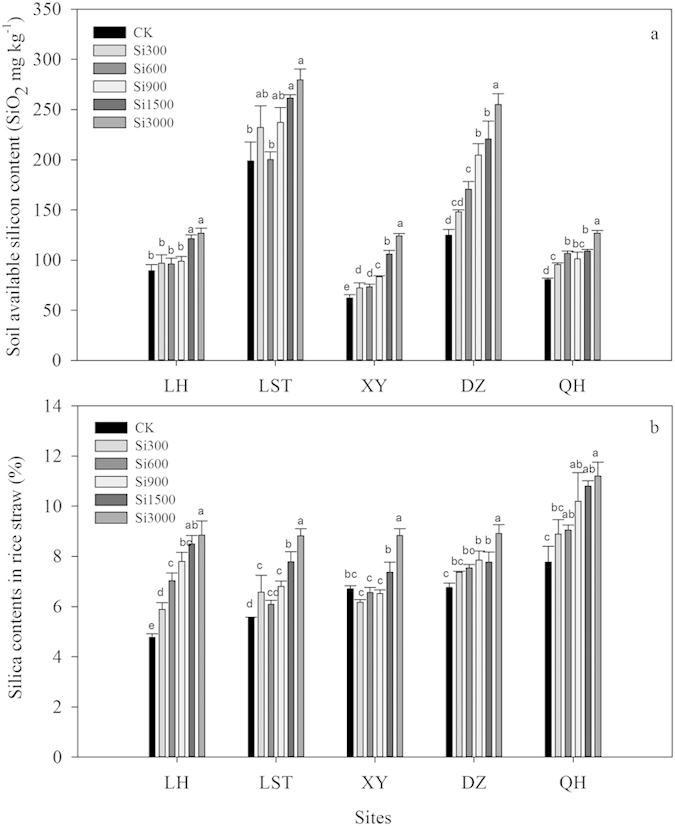
Effect of silicate fertilizer application rate on post-harvest soil available Si contents (a) and silica contents in rice straw (b) at the five sites. Data are means of three replicates. Mean values followed by different letters at the same site differ significantly at the (*P *< 0.05) level of significance.

Post-harvest soil Si concentrations at the five sites tended to increase with increasing silicate fertilizer application rates (Fig. 1a). At Longhui (LH) and Lengshuitan (LST), soils receiving the highest silicate fertilizer application rates (Si1500 and Si3000) also had highest post-harvest Si levels. At Xiangyin (XY) and Qionghai (QH), soil Si concentrations tended to increase with increasing silicate fertilizer application rates. At Danzhou (DZ), positive effects of silicate fertilizer were seen at all application rates above 300 kg ha^−1^.

### Silica contents in rice straw

Silica concentrations in rice straw (leaf and stem) increased with increasing silicate fertilizer application rates (Fig. 1b). At LH, the silica concentrations of rice straw were 23%, 47%, 63%, 78% and 85% higher for the Si300, Si600, Si900, Si1500 and Si3000 treatments, consecutively, when compared with the non-treated control (CK), and 19%, 10%, 23%, 41% and 59% at LST, consecutively. At XY, DZ and QH sites, straw silica concentrations were highest at the Si3000 application rate. At DZ and QH, rice straw silica concentrations were higher than those of the controls at all rates above 600 kg ha^−1^ and 300 kg ha^−1^, respectively.

### Phytolith and PhytOC contents in rice straw

**Figure 2:**
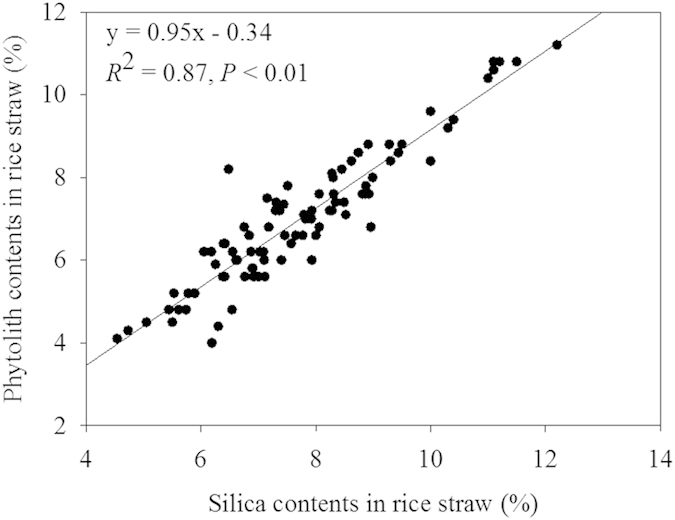
Correlation between phytolith contents (%) and silica contents in rice straw (%) at the five sites tested (*P* < 0.01, n = 90).

The percent phytolith contents in rice straw at the five sites ranged from 4.30% to 10.40% (Table 1), with the lowest levels recorded for the LH CK plots, and the highest levels recorded for the QH Si3000 plots. A trend was seen in increasing phytolith concentrations with increasing rates of silicate fertilizer applications (Table 1). There was also a significant correlation between the silica contents in rice straw and the phytolith contents in rice straw (Fig. 2).

The percentages of C contents in phytoliths ranged from 2.11% to 4.59% at the five sites tested and tended to decrease with increasing rice straw silica contents (Table 1). However, the PhytOC contents, based on percent straw dry weight, varied from 0.12% to 0.46% and tended to increase with increasing silicate fertilizer application rates (Table 1).

### Estimated ANPP, phytolith production flux, phytolith production rate, PhytOC production flux, and PhytOC production rate

**Figure 3:**
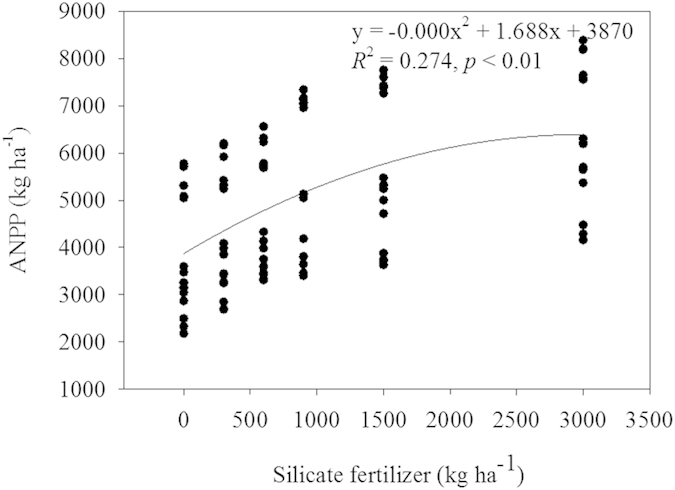
Correlations between Si fertilizer rate (kg ha^−1^) and ANPP (kg ha^−1^) at the five sites tested (*P* < 0.01, n = 90).

ANPP, phytolith production flux, phytolith production rate, PhytOC production flux and PhytOC production rate are shown in Table 2. ANPP at the five sites ranged from 2334.90 to 8256.27 kg ha^−1^ yr^−1^, with the lowest levels recorded for the DZ CK plots and the highest levels recorded for the LST Si3000 plots (Table 2). A trend was seen in increasing ANPP with increasing silicate fertilizer application rates (Fig. 3).

The phytolith production flux and the phytolith production rate at the five sites ranged from 129.95 to 677.40 kg ha^−1^ yr^−1^ and 3.91 × 10^6^ tonnes yr^−1^ to 20.39 × 10^6^ tonnes yr^−1^, respectively (Table 2). The lowest rates were recorded for the LH CK plots, whereas, the highest were recorded for the LST Si3000 plots. The change in phytolith production flux and the phytolith production followed a similar rate-dependent trend as ANPP, and once again, the highest recorded levels at each site were from the Si3000 treated plots.

The PhytOC production flux and the PhytOC production rates, varying greatly from 16.24 (DZ CK plots) to 104.24 (QH Si3000 plots) kg CO_2_ ha^−1^ yr^−1^ and 0.49 × 10^6^ (DZ CK plots) to 3.14 × 10^6^ (QH Si3000 plots) tonnes CO_2_ yr^−1^, respectively, were always highest at the Si3000 rate at each site tested (Table 2).

### Correlation analysis

**Figure 4:**
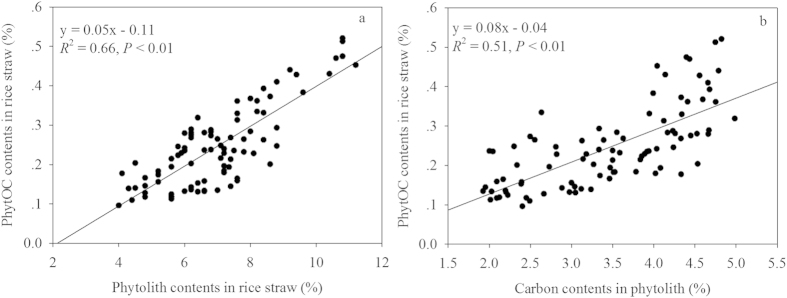
Correlations between phytolity contents in rice straw (%) and PhytOC contents in rice straw (%) (a) and carbon contents in phytolith (%) with PhytOC contents in rice straw (%) (b), at the five sites tested (*P* < 0.01, n = 90).

**Figure 5:**
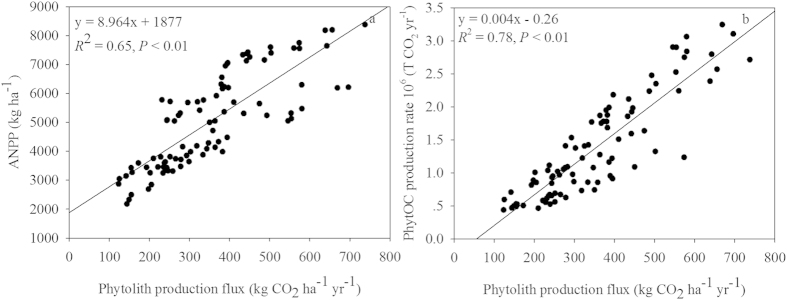
Correlations between ANPP and phytolith production flux (a) and PhytOC production rate with phytolith production flux (b) in rice straw at the five sites tested (*P* < 0.01, n = 90).

The relationships were examined among phytolith contents, carbon contents in phytolith, PhytOC contents in rice straw, ANPP, phytolith production flux and PhytOC production rate (Fig. 4). The results show that there are strong positive correlations between the contents of phytolith in rice straw and the PhytOC contents in rice straw (*R*^2^ = 0.66, *P* < 0.01) (Fig. 4a), and between the carbon contents of straw phytoliths and the PhytOC contents in rice straw (*R*^2^ = 0.51, *P* < 0.01) (Fig. 4b). In addition, phytolith production flux was positively correlated with either ANPP (Fig. 5a) or PhytOC production rate (Fig. 5b).

## Discussion

### Factors controlling rice growth and silicon uptake for phytolith production

Plants differ greatly in their ability to accumulate Si, with dry weight concentrations ranging from 0.02% to 32% SiO_2_^27^,^30^. Paddy rice is categorized as a Si accumulating crop having straw contents of 10% to 20% SiO_2_^31^. The differences in Si accumulation among plant species is closely tied to the ability of the plant’s root system to take up [Si(OH)_4_]^27^. However, variations in response to silicate fertilizer additions in rice cropping systems have been attributed to the soil’s native soluble Si content, soils with low to medium native soluble Si content exhibiting a greater response than soils with high native soluble Si content^32^. The variability in rice silica uptake in this trial attributed to the soluble silicon supplying capacity of each soil is shown in Fig. 1a. As this graph represents post-harvest soil levels, these values would include initial soil silicon levels, plus any silicate additions, minus plant removal. The QH site might be more efficient at supplying soluble Si to meet the plant’s needs during the 2012 rice cropping season. At the XY, DZ, and QH sites, the Si3000 silicate fertilizer treatment rate had higher residual levels than the other treatments. The LST site had the highest native soluble silicon levels as evidenced by the CK plots, but the fact that the highest rates of silicate fertilizer (Si1500 and Si3000) resulted in higher post-harvest soil soluble Si and also increased straw SiO_2_ levels over the CK plots (Fig. 1) suggests a more efficient soluble Si supplying capacity from the silicate fertilizer material than from native soil supplies. And, considering that in many areas of China under rice crop production systems, depletion of plant-available Si from soils has been linked to decreased rice yields^25^, we agree that the soil test critical levels at which silicate fertilizer additions are recommended need further research.

Plant tissue phytoliths perform a beneficial function by increasing structural rigidity and mechanical strength^33^,^34^,^35^,^36^. Silica phytoliths also enhance the plants’ resistance to biotic and abiotic stresses^27^,^37^. The beneficial effects of silicon nutrition in rice and other crop production systems, globally, are not new, but have been reported extensively^25^,^33^,^38^,^39^,^40^,^41^. In this study, rice straw Si uptake (Fig. 1b), and dry weights (Table 2) were increased by the different rates of silicate fertilizer addition, supporting the previously referenced research of the beneficial effects of Si nutrition on rice growth.

In the present study, the percentage of plant silica phytolith contents were increased with increasing application rates of silicate fertilizer, with the highest application rate (Si3000) consistently increasing the phytolith contents by 31.85 to 85.20% for all the five sites above control levels (Fig. 2, Table 1), and also increasing the PhytOC percentage in the straw for the five sites tested (Table 1). A recent study indicated that the additions of mulch with high phytolith contents would further increase bamboo soil PhytOC accumulation, providing effective long-time storage of organic C, and significant mitigation potential for climate change^29^. Their research supports the need for Si fertilization as they stated that the increased PhytOC storage in the top soil layer resulted from the accumulation of phytoliths rather than the concentrations of C in the phytoliths. They also mentioned the potential for Si fertilization to indirectly increase PhytOC storage through increased litter-fall. However, research on the effects of Si fertility in increasing PhytOC production was not evaluated in their trial. As such, soil applications of silicate fertilizers have the potential to not only supply Si for plant uptake and deposition in rice straw (Fig. 1) to meet production needs, but also increase the phytolith contents and the PhytOC contents in the straw (Table 1).

### The role of rice in the global phytolith production and the potential of carbon occlusion within phytoliths.

In this study, ANPP values, estimated phytolith production flux, and PhytOC production flux values for rice straw at the five sites were all consistently higher with the Si3000 rate application when compared with all other treatments, and trends were seen in increasing levels with increasing rates of silicate fertilizer additions overall (Table 2). The above-ground phytolith production rate during rice production in China, based on the CK plot data from our five rice cropping sites, would amount to between 3.91 × 10^6^ and 10.94 × 10^6^ tonnes yr^−1^, with no silicate fertilizer additions. Currently, the total estimated area of rice production in China amounts to 30.1 × 10^6^ ha^42^. If the variety of rice grown at XY, *Jinyou277*, were to be grown at all the rice paddy sites across China, this change alone has the potential to increase the above-ground PhytOC production and carbon sequestration rate to approximately 0.94 × 10^6^ tonnes CO_2_ yr^−1^, with no additional silicate fertilizer inputs. However, the widespread use of only one grain variety can have devastating effects, due to genetic uniformity. In the United States during the years 1969 and 1970, 85% of US corn production acreage was affected by a Southern corn leaf blight (causal agent, *Bipolarismaydis* race T) epidemic, due to the extensive planting of a single hybrid corn (*Zea mays* L.) variety carrying the *cms-T* male sterility gene^43^. Therefore, a more viable option of increasing CO_2_ sequestration during rice paddy production is with silicate fertilizer additions. From our trial, CO_2_ sequestration could be increased, on the average, by 67.83%, or 0.16 to 2.17 × 10^6^ tonnes CO_2_ yr^−1^ with silicate fertilizer additions.

Research on phytolith production and phytolith occluded carbon has recently received the attention of other researchers working with various crops. The PhytOC sequestration rates for millet^13^, wheat^44^, sugarcane^14^, bamboo^10^ and rice^8^ have been estimated at 0.03, 0.25, 0.36, 0.70 and 0.13 tonnes CO_2_ ha^−1^ yr^−1^, respectively. These results reveal how agricultural production activities can affect the overall global C balance. More interesting is the fact that all of the above-mentioned crops are known to be Si accumulators, and have been shown to benefit from silicon fertility^45^,^46^,^47^,^48^. So, the potential to expand CO_2_ sequestration beyond China’s paddy rice production, and to increase CO_2_ sequestration levels with silicate fertilizer additions, worldwide, is huge.

However, the need for our current focus on China’s rice paddy production systems is intimately tied to human food security^49^. Of particular concern are the multiple cropping indexes, which have been increasing recently in Southeast Asia and particularly in China during recent years. To the best of our knowledge, our results are the first field evidence showing that such agricultural management practices as silicate fertilizer additions during the rice growing season can potentially increase the storage of PhytOC, an important soil C fraction with long-term stability.

These effects of silicate fertilizer on PhytOC contents appear to be both direct and indirect, by improving PhytOC contents in rice straw and plant growth (Figs 3 and 4a, Table 1), which increased phytolith accumulation (Table 1). In previous studies, supplemental Si under heavy-nitrogen inputs affected improvements in rice yields and grain quality^20^. In this study, we determined that a significant positive correlation existed between the phytolith and silica contents of rice straw with application of silicate fertilizer (*R*^2^ = 0.87, *P* < 0.01; Fig. 2). Strong positive correlations were also exhibited between PhytOC content, based on straw dry weight, and phytolith or carbon content in phytolith (Fig. 4a,b). These results suggest that PhytOC concentrations based on dry weight are closely related to, not only phytolith content, but also the efficiency of C deposition during phytolith formation in rice plants following silicate fertilizer additions. Our results are in agreement with the previous work of Li *et al.*^8^ on rice, that the phytolith content and the PhytOC content based on dry weight correlated well. Although some researchers have suggested that the PhytOC contents of bamboo, wheat, sugarcane and millet are not directly tied to actual phytolith content^10^,^13^,^14^,^41^ factors such as variety, disease resistance, and nutrient requirements can play a role in phytolith accumulation during plant growth^12^,^32^,^44^. In this study, phytolith concentrations were increased with increasing Si fertilizer rate at the five different sites with each site having a different rice variety. However, the highest rate of silicate fertilizer additions proved to be the most effective at all the sites and for all the varieties tested (Table 1, Table 3).

With the increasing effects of global climate change on agriculture in the developing world^50^,^51^, fertility measures will undoubtedly become more and more important in sustaining and improving crop yields and grain quality. Therefore, in the future, silicate fertilizers may be targeted not only for their beneficial effects in increasing stress resistance and yields, but also for their indirect effects in increasing the rate of atmospheric CO_2_ sequestration. Moreover, PhytOC is very stable and can accumulate and remain in soils for hundreds and thousands of years after plant decomposition^7^,^12^. Relative to other forms of organic carbon, the PhytOC is considered to be an important part of the stable organic C fraction in soil. For example, *in situ* decomposition at Numundo sites in Australia showed PhytOC representing up to 82% of the total soil C was buried in soils up to a 2 m depth, whereas the concentration of the total C decreased markedly over a period of 2000 years^7^. PhytOC is highly resistant to decomposition when compared to other organic C components in the soil environment. By using a silicate fertilizer to increase rice growth and ANPP (Fig. 3), we may fully realize the long-term C sequestration and mitigation of global climate change potential. For the five sites receiving different application rates of silicate fertilizer, a strong and positive relationship existed both between ANPP and the phytolith production flux, and between the PhytOC production rate and phytolith production flux (Fig. 5). These results suggest that ANPP, at least for rice, is instrumental in determining the above-ground phytolith production flux and PhytOC production rate (Table 2). And, silicate fertilization practices may enhance ANPP and PhytOC production rate of rice plants. Improving the PhytOC production rate during rice production in China by optimizing Si supply (Si3000 application rate) during the growing period may provide a sustainable and important means for crop production to reduce atmospheric carbon buildup, thus decreasing CO_2_ induced global climate change.

## Conclusions

Our study reveals that addition of silicate fertilizer affects PhytOC sequestration by improving PhytOC contents in rice straw and the plants growth; the average above-ground phytolith-occluded carbon (PhytOC) production rates at five of China’s paddy rice production areas are increased by the addition of silicate fertilizer. Our results are the first field evidence that there is a potential to increase the storage of PhytOC through such agricultural management practices as the addition of silicate fertilizer. Therefore, regulating Si supply during rice growth may serve as an effective tool in improving PhytOC production rate and play an important role in carbon bio-sequestration and global warming mitigation.

## Methods

### Experimental design

Field experiments were conducted simultaneously at 5 different paddy rice field sites across China during the 2012 growing season. The 5 selected sites included Longhui (LH, E110°51′19.2″, N27°07′45.3″, elevation 228.3 m), Lengshuitan (LST, E111°35′30.8″, N26°36′59.1″, elevation 142 m) and Xiangyin (XY, E112°52′9.5″, N28°36′27.4″, elevation 228.3 m) in Hunan povince, and Danzhou (DZ, E109°30′14.0″, N19°30′17.6″, elevation 135 m) and Qionghai (QH, E110°27′39.8″, N19°20′48.1″, elevation 60.0 m) in Hainan povince. All the consecutive trial plots remained consistent for treatment as experiments initiated in 2011. The basic information of the five sites is listed in Table 3.

There were six treatments based on silicate fertilizer rates, i.e. Control (CK), 300 (Si300), 600 (Si600), 900 (Si900), 1500 (Si1500) and 3000 (Si3000) kg ha^−1^ with 3 replicates of each treatment. In total, 18 plots (5 × 6 m each) were arranged in a randomized complete block design at each site. The silicate fertilizer was applied as the basal fertilizer one week prior to rice seedling transplant.

### Soil and plant sampling

Soil samples were taken from the top layer (0–20 cm) from five randomly-selected positions within each plot after treatment application of silicate fertilizer. The soil samples were thoroughly mixed and sieved to pass a 2.0 mm mesh screen. A 1 kg subsample of soil was sealed in air-tight bags prior to laboratory submission. All soil samples were air dried prior to analysis for plant-available Si contents.

Plant subsamples were collected at rice grain maturity. Five rice traps (100 cm × 100 cm in size) were randomly placed on the rice floor of each plot during harvest. The samples were washed carefully with running water followed by repeated rinsings with distilled water. The cleaned samples were oven-dried to a constant weight at 80 °C for 72 h, then ground to pass a 0.15 mm sieve prior to analysis of silica content, phytolith content, and PhytOC content.

### Chemical analysis

Plant-available Si in the soil was determined using the pH 4.0 acetate buffer solution method that includes a 40 °C for 5 h setting period followed by silicon molybdenum blue spectrophotometry analysis^52^.

Silica content in rice straw (leaf and stem) was determined by the colorimetric Si molybdenum blue method^53^. Briefly, 0.1 g pretreated plant samples are fused using the high-temperature alkaline fusion method then diluted to 50 ml with distilled water, followed by colorimetric molybdenum blue analysis^53^.

Phytolith extraction from leaf and stem tissue was accomplished using microwave digestion as described by Parr *et al.*^54^. This process was followed by Walkley-Black digestion^55^ to ensure removal of any potentially extraneous organic materials. The phytolith extraction solution was also used to examine for extraneous organic materials outside the phytolith cells by adding 0.8 mol L^−1^ potassium dichromate to the solution. If the color of the solution remained unchanged for 5 min, this was an indicator that any extraneous organic materials outside the phytoliths had been thoroughly removed. The phytoliths extracted were oven-dried at 75 °C for 24 h in a centrifuge tube of known weight. The samples were allowed to cool and then weighed with the tube weight subtracted to obtain the phytolith quantities.

Carbon content of phytoliths (PhytOC) was determined using a CNS (carbon, nitrogen, sulfur) Analyzer (Elementar Americas Inc., Mt. Laurel, NJ USA).

Straw dry weight was determined using the weight of five rice traps (100 cm × 100 cm in size) within each plot. The traps were randomly placed within each plot during harvest. The samples were oven-dried to a constant weight at 80 °C for 72 h, and then weighed to obtain the straw dry weight after removal of rice grains.

### Construction of silica-phytolith content transfer function

The silica-phytolith content transfer function was constructed using the regression analysis method based on the determined silica and phytolith contents of the rice straw samples (Fig. 2). Silica content was converted to phytolith content using the following Equation:


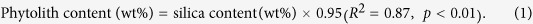


### Estimation of phytolith and PhytOC production flux and rate

As the production of biogenic Si is primarily driven by plant Si concentration and ANPP^56^, the phytolith production flux of rice aboveground biomass was estimated using rice straw phytolith content data (Equation 1, above) and ANPP as shown below in Equation 2:


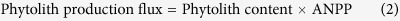


Where:

Phytolith production flux is the total phytolith production in rice aboveground biomass per ha per year (kg ha^−1^ yr^−1^).

Phytolith content is the content of phytoliths in rice aboveground biomass (wt%).

ANPP is aboveground net primary productivity of rice (kg ha^−1^ yr^−1^)^41^.

Phytolith production rate of rice aboveground biomass was estimated using the data from the phytolith production flux and the rice straw sampling area as shown in Equation 3, below:


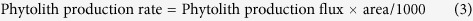


Where:

Phytolith production rate is total phytolith production of rice aboveground biomass per year (T yr^−1^),

Phytolith production flux is obtained from Equation (2) and,

Area is the area of rice planting (10^6^ ha).

PhytOC production flux of rice aboveground biomass was determined using the phytolith production flux and PhytOC content of phytoliths data as shown in Equation 4, below:


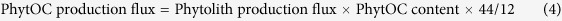


Where:

PhytOC production flux is the total PhytOC production in rice straw per area per year (kg CO_2_ ha^−1^ yr^−1^);

Phytolith production flux is obtained from Equation (2);

PhytOC content is the content of PhytOC in the phytoliths.

PhytOC production rate of rice aboveground biomass was obtained from the PhytOC production flux and rice area data as shown in Equation 5:


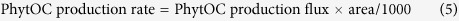


Where:

PhytOC production rate is the total PhytOC production of rice by rice straw (T CO_2_ yr ^−1^);

PhytOC production flux is obtained from Equation (4) and,

Area is the area of rice planting (10^6^ ha).

### Statistical analysis

All the experimental data presented in this paper were statistically examined by one-way analysis of variance between the means of different treatments at the same rice growing site. Before all data analysis, the normality of data was tested by Shapiro-Wilk test according to a significance level at *P* < 0.05 under the null hypothesis that the population is normally distributed, Shapiro-Wilk tests indicated that most of the data met the normality test, while for the datasets that showed non-normality, a log-transformation was performed prior to ANOVA analysis using Duncan’s multiple range test. Similar test results were obtained when using either the original or transformed data, so, the authors opted to present the results using the original data. Statistical significance of the means of three replicates was compared at the 0.05 probability level using SAS (Version 8). All figures were drawn using SigmaPlot software (12.5) or Excel 2013.

## Additional Information

**How to cite this article**: Song, A. *et al.* The potential for carbon bio-sequestration in China's paddy rice (*Oryza sativa* L.) as impacted by slag-based silicate fertilizer. *Sci. Rep.* **5**, 17354; doi: 10.1038/srep17354 (2015).

## Figures and Tables

**Table 1:** Experimental sites, Percentage of phytolith contents, percentage of carbon contents in phytolith and percentage of PhytOC content in straw.

Site	Treatment	Percentage of phytolith contents (%)	Percent carbon contents in phytoliths (%)	Percent PhytOC in straw (%)
LH	CK	4.30 ± 0.12d	4.03 ± 0.40a	0.17 ± 0.02b
	Si300	4.97 ± 0.33cd	3.33 ± 0.25ab	0.17 ± 0.02b
	Si600	5.60 ± 0.46bc	2.35 ± 0.19cd	0.13 ± 0.01b
	Si900	6.73 ± 0.47ab	2.11 ± 0.05d	0.14 ± 0.01b
	Si1500	7.73 ± 0.47a	2.14 ± 0.12d	0.17 ± 0.02b
	Si3000	7.60 ± 0.40a	3.06 ± 0.18bc	0.23 ± 0.02a
LST	CK	4.93 ± 0.13cd	3.09 ± 0.32ab	0.15 ± 0.02c
	Si300	5.47 ± 0.27c	3.65 ± 0.13a	0.20 ± 0.02b
	Si600	4.53 ± 0.35cd	2.63 ± 0.19bc	0.12 ± 0.02c
	Si900	5.60 ± 0.00c	2.15 ± 0.04c	0.12 ± 0.00c
	Si1500	6.73 ± 0.35b	2.18 ± 0.13c	0.15 ± 0.01c
	Si3000	8.20 ± 0.31a	3.43 ± 0.06a	0.28 ± 0.01a
XY	CK	6.53 ± 0.84b	3.91 ± 0.27bc	0.26 ± 0.05a
	Si300	6.27 ± 0.07b	4.59 ± 0.20a	0.29 ± 0.02a
	Si600	6.00 ± 0.12b	4.52 ± 0.14ab	0.27 ± 0.01a
	Si900	6.30 ± 0.26b	3.77 ± 0.27c	0.24 ± 0.02a
	Si1500	6.27 ± 0.27b	4.07 ± 0.09abc	0.26 ± 0.02a
	Si3000	7.80 ± 0.31a	3.97 ± 0.21abc	0.31 ± 0.02a
DZ	CK	6.40 ± 0.12b	2.11 ± 0.09e	0.14 ± 0.00d
	Si300	7.25 ± 0.05ab	2.56 ± 0.04d	0.19 ± 0.00c
	Si600	7.43 ± 0.20a	2.97 ± 0.05c	0.22 ± 0.01b
	Si900	7.33 ± 0.44ab	3.96 ± 0.09a	0.29 ± 0.01a
	Si1500	7.07 ± 0.26ab	3.41 ± 0.14bc	0.24 ± 0.00b
	Si3000	7.90 ± 0.59a	3.04 ± 0.23bc	0.24 ± 0.01b
QH	CK	6.93 ± 0.55b	4.46 ± 0.10a	0.31 ± 0.03b
	Si300	8.53 ± 0.58ab	4.47 ± 0.24a	0.38 ± 0.01ab
	Si600	8.33 ± 0.37ab	4.37 ± 0.16a	0.37 ± 0.03ab
	Si900	9.60 ± 1.20a	4.25 ± 0.54a	0.42 ± 0.10ab
	Si1500	10.13 ± 0.37a	4.38 ± 0.12a	0.44 ± 0.01a
	Si3000	10.40 ± 0.61a	4.41 ± 0.22a	0.46 ± 0.01a

Data are means of three replicates. Mean values followed by different letters in the same site are significantly different (P < 0.05).

**Table 2:** The estimated ANPP, phytolith production flux, phytolith production rate, PhytOC production flux and PhytOC production rate per year in T of CO_2_.

Site	Treatment	Straw dry weight (ANPP) (kg ha^−1^ yr^−1^)^1^	Phytolith production flux (kg ha^−1^ yr^−1^)^2^	Phytolith production rate (10^6^ tonnes yr−1)^3^	PhytOC production flux (kg CO_2_ ha^−1^ yr^−1^)^4^	PhytOC production rate (10^6^ tonnes CO_2_ yr^−1^)^5^
LH	CK	3021 ± 82e	130 ± 6e	3.91 ± 0.18e	19.34 ± 2.58bc	0.58 ± 0.08bc
	Si300	3382 ± 55de	168 ± 12de	5.06 ± 0.37de	20.75 ± 3.19bc	0.62 ± 0.10bc
	Si600	3599 ± 86cd	201 ± 15d	6.06 ± 0.44de	17.23 ± 1.13c	0.52 ± 0.03c
	Si900	3933 ± 126cd	266 ± 27c	8.00 ± 0.81c	20.50 ± 1.94bc	0.62 ± 0.06bc
	Si1500	4531 ± 337b	347 ± 7b	10.45 ± 0.22b	27.25 ± 1.27b	0.82 ± 0.04b
	Si3000	5572 ± 103a	424 ± 27a	12.75 ± 0.79a	47.74 ± 4.70a	1.44 ± 0.14a
LST	CK	5069 ± 11f	250 ± 6d	7.53 ± 0.19d	28.36 ± 3.26c	0.85 ± 0.10c
	Si300	5329 ± 51e	292 ± 17d	8.78 ± 0.51d	39.20 ± 3.76b	1.18 ± 0.11b
	Si600	5729 ± 27d	260 ± 19d	7.81 ± 0.57d	25.29 ± 3.69c	0.76 ± 0.11c
	Si900	7016 ± 32c	393 ± 2c	11.83 ± 0.05c	30.91 ± 0.44bc	0.93 ± 0.01bc
	Si1500	7540 ± 145b	509 ± 36b	15.31 ± 1.08b	40.46 ± 2.27b	1.22 ± 0.07b
	Si3000	8256 ± 63a	677 ± 31a	20.39 ± 0.92a	85.00 ± 3.13a	2.56 ± 0.09a
XY	CK	5597 ± 145e	363 ± 36c	10.94 ± 1.09c	52.80 ± 9.01b	1.59 ± 0.27b
	Si300	6095 ± 89d	382 ± 9c	11.50 ± 0.26c	64.38 ± 4.25b	1.94 ± 0.13b
	Si600	6370 ± 98c	382 ± 2c	11.50 ± 0.07c	63.36 ± 2.16b	1.91 ± 0.06b
	Si900	7208 ± 65b	454 ± 17b	13.66 ± 0.50b	63.01 ± 6.22b	1.90 ± 0.19b
	Si1500	7405 ± 12ab	464 ± 20b	13.97 ± 0.59b	69.34 ± 4.41ab	2.09 ± 0.13ab
	Si3000	7592 ± 28a	592 ± 25a	17.83 ± 0.76a	86.28 ± 5.90a	2.60 ± 0.18a
DZ	CK	2335 ± 184e	149 ± 3c	4.49 ± 0.10c	16.24 ± 0.29d	0.49 ± 0.01d
	Si300	2928 ± 68d	212 ± 11b	6.39 ± 0.33b	30.82 ± 1.85b	0.93 ± 0.06b
	Si600	3356 ± 320d	249 ± 5b	7.51 ± 0.14b	31.20 ± 1.72b	0.94 ± 0.05b
	Si900	3501 ± 307c	257 ± 21b	7.75 ± 0.62b	24.22 ± 2.32c	0.73 ± 0.07c
	Si1500	3694 ± 95b	261 ± 11b	7.86 ± 0.34b	20.27 ± 1.42 cd	0.61 ± 0.04 cd
	Si3000	4307 ± 44a	341 ± 32a	10.27 ± 0.97a	37.58 ± 1.50d	1.13 ± 0.05a
QH	CK	3708 ± 92e	239 ± 22c	7.20 ± 0.66c	39.13 ± 3.99c	1.18 ± 0.12c
	Si300	3972 ± 166d	339 ± 26c	10.22 ± 0.78c	55.28 ± 2.30bc	1.66 ± 0.07bc
	Si600	4772 ± 37c	346 ± 22c	10.42 ± 0.66c	55.66 ± 5.05bc	1.68 ± 0.15bc
	Si900	5686 ± 72bc	488 ± 62b	14.68 ± 1.87b	78.44 ± 8.03ab	2.36 ± 0.54ab
	Si1500	5358 ± 33b	542 ± 26ab	16.32 ± 0.78b	86.88 ± 3.76a	2.62 ± 0.11a
	Si3000	6248 ± 93a	648 ± 35a	19.52 ± 1.06a	104.24 ± 1.83a	3.14 ± 0.06a

^1^ANPP is above-ground net primary productivity.

^2^Phytolith production flux was estimated using Equation 2.

^3^Phytolith production rate was estimated using Equation 3.

^4^PhytOC production flux was estimated using Equation 4.

^5^PhytOC production rate was estimated using Equation 5.

**Table 3:** The basic information of the five sites tested.

Site	Rice variety	Geographical conditions	Basal and topdressing fertilization	Transplanting and harvesting time, plant protection and irrigation	Total N (%)	Total P (%)	Available K (mg kg^−1^)	Organic carbon (g kg^−1^)	pH
LH	Tianyouhuazhan	Subtropical monsoon climate zone with effective accumulative temperature of 5081 °C, mean annual precipitation of 1340 mm and mean annual temperature of 16.1–17.1 °C.	Basal fertilizers were applied on 23^rd^ July at a rate of 195 kg-0 kg-67.5 kg ha^−1^ (N-P_2_O_5_-K_2_O). N, P and K were applied in the forms of urea, calcium superphosphate and potassium chloride, respectively, with a basal to topdressing ratio of 7:0:3. Topdressing was done on 2^nd^ August at tillering stage.	The sowing and harvesting time was on 22^nd^ June and 19^th^ October, respectively. Pesticides were sprayed on 12^th^ August and 19^th^ September to control leafroller, stem borer, planthoppers and sheath blight. Irrigation was applied on 30^th^ July, 5^th^, 12^th^ August, 10^th^ September and 2^nd^ October, respectively, to maintain a 5.0 cm water layer above ground.	2.34 ± 0.00	0.35 ± 0.01	171.40 ± 1.78	27.02 ± 0.48	6.02 ± 0.03
LST	T-you207	Subtropical monsoon climate zone with its mean annual precipitation of 1354.6 mm and mean annual temperature of 16.7 °C.	Basal fertilizers were applied on 18^th^ July at a rate of 450 kg ha^−1^ in the form of compound fertilizer (N: P_2_O_5_: K_2_O = 15:15:15). Topdressing fertilizers were applied at a rate of 180 kg ha^−1^ in the form of urea on 10^th^ August 2012.	Rice was transplanted on 23^rd^ July and harvested on 19^th^ October. Pesticides were applied on 13^th^ August, 24^th^ August, 9^th^ September and 19^th^ September, respectively, to control leafroller, stem borer, planthoppers and sheath blight. Irrigation was applied on 18^th^ August and 20^th^ September, respectively, to maintain a 5.0 cm water layer above ground.	2.59 ± 0.02	0.49 ± 0.02	160.48 ± 2.75	32.42 ± 0.66	6.02 ± 0.05
XY	Jinyou277	Subtropical monsoon climate zone with its effective accumulative temperature of 5081 °C, mean annual precipitation of 1340 mm and mean annual temperature of 16.1–17.1 °C.	Basal fertilization was applied on 14^th^ July at a rate of 210 kg-375 kg-0 kg ha^−1^ (N-P_2_O_5_-K_2_O); nitrogen, phosphorus and potassium were applied in the forms of urea, calcium superphosphate and potassium chloride. Topdressing fertilizers were applied at the rates of 90 kg-0 kg-112.5 kg ha^−1^ (N-P_2_O_5_-K_2_O) at tillering stage on 23^rd^ July.	Rice was transplanted on 15^th^ July and harvested on 12^nd^ November. Pesticides were applied on 5^st^ August and 17^th^, 28^th^ September to control leafroller, stem borer, planthoppers and sheath blight. Irrigation was made to rice on 21^st^, 27^th^ July, 9^th^, 29^th^ August, 15^th^, 27^th^ September and 11^st^, 24^th^ November, respectively.	2.23 ± 0.03	0.50 ± 0.05	130.55 ± 0.41	37.62 ± 1.79	5.30 ± 0.01
DZ	Bo II you 312	Tropical monsoon climate zone with its mean annual precipitation of 1500–2000 mm, and mean annual temperature of 23.3 °C.	Basal fertilizers were applied on 22^nd^ August at a rate of 1200 kg ha^−1^ in the form of compound fertilizer (N: P_2_O_5_: K_2_O = 15:15:15), while topdressing fertilizers were applied at a rate of 1200 kg ha^−1^ in the form of compound fertilizer (N: P_2_O_5_: K_2_O = 15:15:15) on 9^th^ September 2012 at tillering stage.	Rice was transplanted on 12^nd^ August and harvested on 19^th^ November. Pesticides were applied on 27^th^ August and 11^th^ September to control leafroller, stem borer, planthoppers and sheath blight. Irrigation was made to rice on 22^nd^ August, 27^th^ August and 22^nd^ September, respectively.	1.00 ± 0.02	0.47 ± 0.01	71.78 ± 1.73	32.09 ± 1.25	5.28 ± 0.03
QH	Tecanzhan 25	Tropical monsoon and marine humid climate zone with its mean annual precipitation of 2000 mm and mean annual temperature of 24 °C.	Basal fertilizers were applied on 15^th^ June at a rate of 112.5 kg ha^−1^ in the form of urea. Topdressing fertilizers were applied at a rate of 150 kg ha^−1^ in the form of compound fertilizer (N: P_2_O_5_: K_2_O = 16:16:16) on 25^th^ June 2012.	Rice was transplanted on 9^th^ June and harvested on 15^th^ September. Pesticides were applied on 1^st^ July, and 15^th^ July to control leafroller, stem borer, planthoppers and sheath blight. Irrigation was made to rice on 22^nd^ June, 27^th^ June and 22^nd^ August, respectively.	1.37 ± 0.02	0.33 ± 0.03	81.70 ± 3.87	19.52 ± 0.64	5.69 ± 0.01
